# What is disability history the history *of*?

**DOI:** 10.1111/hic3.12813

**Published:** 2024-06-11

**Authors:** Coreen Anne McGuire

**Affiliations:** Durham University, Durham, UK

## Abstract

This article has two connected aims. First, to contour the boundaries of modern disability history through outlining its development and second, to provide a new methodological agenda for disability history. The design model of disability has outlined an important new programme to integrate the social and medical models of disability by foregrounding materials. Yet ‘disability things’ (to use Ott's memorable term) have been part of disability history's genesis since the material turn, which started the process of social historians recovering the lives of those not recorded in textual sources through objects, including prosthetics. From considering objects as things, the influence of Science and Technology Studies scholars pushed disability historians to further consider objects as agents and objects in use. These approaches have highlighted the differential levels of autonomy and power that objects and their users have in making history. However, this focus on materials has highlighted visible and recorded disability over ‘invisible’ disability, which has perpetuated its opacity and created definitional difficulties around disability demarcation. Medical history methodologies aimed at revealing the ‘patient view’ can help bring people back into focus but uphold the categories of patients and biomedicine in a way that impedes the aims of disability scholars. Focusing on exactly *what* is hidden is less useful than focusing on *how* it is hidden, and science and technology study methodologies can illuminate these processes.

## 1 Introduction

What disability is for historians depends on the model of disability that they deploy. This is the result of the debt disability historians have to foundational disability studies texts that propounded a social model that explicitly conceptualised disability as the result of forced societal exclusion from the community and the labour market. As it emerged in the late 1980s, the social model separated impairment from disability in a similar way to the sex/gender formation propounded by second-wave feminist scholars. By conceptualising disability as the result of inaccessible structures, it positioned itself in opposition to a medical model which framed disability as the result of individual impairments that could be ‘fixed’ by medical or technical intervention.^1^

The resulting focus on the disabling impact of the environment and the use of technologies to both create and mitigate these effects meant that disability history developed unique strengths in dealing with material culture. This development holds obvious parallels with the rise, some 50 years ago, of histories from below, which focused on the lives of people who were not always literate or documented in traditional textual sources. This ‘material turn’ shifted historians away from documents partly to focus on those lives less recorded and partly in reaction to the semiotic focus of postmodernism, which prioritised analysis of concepts through signs, texts, and discourse.^2^ The material turn challenged historians because textual materials have long been the primary focus of historical work.^3^ The document (including primary sources like parliamentary recordings, birth certificates and wills) is closely linked with authority, legitimacy, and competency—’qualities that are typically associated with nondisabled historical actors’.^4^ Yet despite their seeming ‘truthness’, these kinds of documents, as Ludmilla Jordanova clarifies, ‘are highly mediated; they necessarily pass through the minds of human agents, who select, alter and make mistakes—that is, they transform and translate’.^5^

Engaging with textual documents to write disability history has nevertheless been immensely valuable. Some of the most important formative texts of the field started the recovery of disabled lives by using sources created by the state or by institutions.^6^ However, the close associations between disability and welfare, institutions, and poverty fostered by such approaches can close off investigation of the spaces where disabled people thrived outside of institutions (including at work and in the home) and can de-emphasise the agency and creativity of disabled people.^7^

An important countervailing focus on the working lives of the disabled emerged from the Wellcome-funded ‘Disability and Industrial Society’ project, which ran between 2011 and 2016.^8^ This project focused on the collective disablement of the working classes by investigating disability and coalmining. It revealed that disabled people were not excluded from the labour market during the industrial revolution but worked while disabled and after disablement resulting from this work.^9^ This insight provided an important critique of the uses of history in foundational disability studies texts that propounded a social model that explicitly conceptualised disability as exclusion from the labour market.^10^ Productive combinations of different *types* of textual sources further offset the earlier focus on male industrial disability to produce insights into women's disability experiences (including of chronic illness) in history, for instance through combining state documents like First World War pension records with contemporaneous fiction.^11^

However, what disability historians want to know about disabled experience (how it felt, its contingency, its categorisation) can be difficult to find in written documents, even in combination with methodologies that allow us to read ‘against’ or ‘along’ the grain.^12^ ‘Ego documents’ like letters and diaries, which might contain personal reflections are especially hard to find; partly the result of stigma and intentional concealment, and partly the result of segregation in institutions that discouraged the creation of such documents and sometimes destroyed them if they were produced.^13^

Therefore, despite the empirical errors and conceptual omissions that recent work has highlighted in the first articulations of the social model, disability history remains bound to its focus on the material. The way that technologies have been designed to be inaccessible to the disabled was after all the foundational insight of the social model of disability. The idea that the problems faced by the disabled *wholly* originate in the environment rather than in their individual bodies has been roundly critiqued.^14^ Yet a material focus has been fruitfully integrated into Bess Williamson and Elizabeth Guffey's ‘Design Model of Disability’, which proposes that the experiences, meanings, and definitions of disability are shaped by design.^15^ This is an important new way of engaging with the social model while retaining a focus on design and technologies.

Integrated investigation of medical technology using social model theory has not often been done. The social model created specific difficulties in integrating the history of medicine with disability history because of the implicit association between the study of medical things and the medical model.^16^ As Beth Linker pointed out in her oft-cited 2013 positioning piece, the separation between the two disciplines was in part fuelled by disciplinary world building, ‘the desire among disability historians to define themselves as something other than medical historians’.^17^ The separation of disability from medicine was further perpetuated by the force of the medical/social model binary, which maintained that studying ‘the sites and devices that have been brought to bear on the disabled body’ acted to further perpetuate the medical model.^18^ Defining disabled people outwith the parameters of medicine and patients has been an especially important aspect of disability history because of the influence and commitments of the deaf history. The strongest component of early disability history was Deaf history, which capitalised Deafness to indicate that the Deaf formed a cultural group linked by shared language use separate from deafness as a medical condition.^19^ Pioneering Deaf historians often explicitly separated their work and experiences from disability to locate the disablement they experienced in hearing people's exclusionary practices: ‘So Deaf people generally do not see themselves as disabled nor do they seek what people who say they are disabled seek’.^20^ Deaf history's more recent alignment with the emphasis of ‘the new disability history’ on disability as an analytic category both comparable to categories like race, gender, and class and revealing of these identities has brought Deaf history back into disability history to an extent.^21^ However, medical history and disability history remain at odds.

The abyss between medical history and disability history seems especially hard to traverse for historians of psychiatry. As the introduction to the *Oxford Handbook of Disability History* makes clear, this is partly linked to the perceived positionality and actual subjectivity of the historian,

Where one historian sees a patient, another sees an inmate; where one historian sees a desire to treat and relieve suffering, another sees the desire to control socially deviant behavior; where one historian sees rehabilitation and occupational therapy, another sees the exploitation of a captive labor force; where one historian may focus on violent and abusive patients, another may focus on violent and abusive attendants and sadistic physicians; where one historian sees signs or symptoms of illness or pathology, another sees acts of resistance.^22^

Yet, as Andrew Hogan has emphasised, medical historians have not actually tended to work to uphold the medical model.^23^ The social/medical model binary is in some ways a strawman for historians, though it remains an incredible powerful political tool. Yet even outwith a social/medical model framework, the influence of Foucauldian analysis has meant that medical histories from below have frequently and explicitly focused on medicine as an oppressive force, especially in relation to histories of childbirth and psychiatry.^24^ The focus on the patient as a medical construct rather than an active agent and the reliance on doctors' notes have made it difficult to respond to Porter's call to heed the patient voice and to extend medical history methodologies to those who did not and do not identify as patients.^25^

Despite these metaphysical and methodological difficulties, demarcating science, medicine, and their related histories as oppressive forces working in opposition to the social model prevents real engagement with how precisely science and medicine worked to create disability. The neat separation of disability from impairment means that the conception of impairment is reified as objective; this split thus implicitly supports the legitimacy of the naturalist medical model.^26^ As Hamraie has argued, we are missing ‘a broader understanding of the epistemological status of knowledge-claims about disability’ because within disability studies following such approaches ‘power (particularly biopower), rather than knowledge’ has been the unifying focus.^27^ As John Warner emphasised in 1995, ‘most historians of medicine never shed a guarded leaning to biological realism’.^28^ In fact, ‘those most squeamish about recognising the material reality of disease have the greatest affinity to history of science’.^29^ We need to develop methodologies capable of analysing the medical model of disability as *social*. Although the design model brings welcome new attention to the importance of technologies, approaches pioneered within the history of science and Science and Technology Studies (STS) can reveal the disabling impacts of science itself and allow us to attend to disability in its broadest sense.

## 2 Section 1: Objects As Agents

Disability historians turned to objects with increased intent after 2002, following the publication of Katherine Ott and David Serlin's *Artificial Parts, Practical Lives*.^30^ This sterling publication emphasised that disability stands apart from the other categories of historical analysis because ‘it is bonded with technology, tools, and machines as a medium of social interaction’.^31^ It centred objects as they related to bodies, and pushed back against postmodernist tendencies to conceptualise prostheses purely as metaphorical or narrative devices. By ‘keeping prostheses attached to people’ this work drew our attention back to the things themselves while highlighting their user's role in personalising and adapting them.^32^ For instance, such revelations came through strongly in Raman Srinivasan's discussion of the racialised and gendered development of the Jaipur Foot, which asked: ‘Who would have thought of putting toe-rings and henna patterns on a prothesis?’.^33^

The material turn in disability history stimulated the conceptualisation of disability beyond the social/medical model binary by identifying it explicitly ‘as a phenomenon that can be treated or ameliorated through digital or material things’.^34^ This approach has uncovered the longer history of the social model by revealing architectural accommodation of disability in ancient Greece through documentation of the more extensive use of stone ramps in healing sanctuaries compared to non-healing sites.^35^ Focusing on materiality shifts our focus onto disability as mediated by the environment and increases evidence of its continuity, both throughout history and in the lives of individuals. As Bess Williamson notes, ‘When it comes to material traces of disability, objects such as canes, splints, and eye patches preserved over time remind us of the persistence of physical variation as well as its social nature’.^36^ Gemma Almond's study of spectacles in the Science Museum collection has additionally demonstrated that evidence of wear and tear can reveal *how much* objects were used by individuals as well as how they were modified.^37^ This kind of close analysis can also reveal the fit, flex, and dynamic function of prosthesis to ‘document movement’ and illuminate how the wearer *moved with* objects.^38^ Other histories, for instance of the wheelchair, have demonstrated how design standards impeded wheelchair user's movements by anticipating the needs of the able-bodied user over those of the disabled user.^39^ Such analysis is not restricted to periods of mass production (indeed, disability things are rarely standardised) and has been successfully deployed in the early modern period; the study of iron hands has revealed the nature of war injuries and the mechanistic qualities of the object necessary to allow their wearers to continue to work.^40^

There is undoubtedly something special about engaging with an object in a hands-on way that ‘gives a tactile, sensory dimension to the past’.^41^ It is, for instance, the only way to get at a very important aspect of prosthetic design—the weight of the object.^42^ There is usually a trade-off between portability and usability when it comes to the weight of prosthetics. Making objects smaller and lighter has been especially desirable historically in the case of prosthetics that made stigmatised disability visible, such as hearing aids and respiratory prosthetics like ambulatory oxygen.^43^ But making such devices smaller often made them less effective. Weight is thus an integral aspect of our understanding of whether prosthetic design was driven by stigma or by power. More than measurement, handling an object delivers affective information; ‘Exploring the experience of users through touch offers unique insights into agency and control—particularly for marginalised groups—by allowing the historian to explore individuals’ emotional attachment or how they adapted or customised their own device’.^44^ Affective experiences can be provoked by objects too, as ‘contact with an object conditions feelings in human subjects’.^45^ Although recovering the feelings and emotions attached to an object is methodologically challenging, Sally Holloway's discussion of the textile objects eighteenth-century mothers left with or created for their abandoned children masterfully shows how emotional meaning could be threaded into materials to be recovered later.^46^

However, it is both possible and effective to investigate objects in terms of their relationship to individuals through a variety of material cultures, including texts and images. For instance, Jaipreet Virdi's exploration of artist Dorothy Brett's relationship with her hearing aid trumpet uses Brett's artwork to explore her changing feelings about her deafness by showing that she positioned her hearing aid in her self-portraits as an integrated part of her body.^47^ Such work on the history of objects considers them not only in terms of access but fundamentally *in relation to their use by people*—the objects are not of interest because of their uniqueness or beauty or totemic qualities but are important because people regarded them as essential components of themselves.^48^

Focus on recovering the agency of disabled people through their object use means that disability historians face an ambivalent task when adopting methods predicated on artefactual agency. Debates about the power and agency of objects within the material culture tradition have been largely bypassed by disability historians for whom the claim that objects create and shape ‘experiences, identities and relationships’ and that ‘humans and things are enmeshed in ways that deny … the division between the social and the material world’ has been so obviously the starting point of any historical investigation into disabled lives.^49^

The literal power of objects and artefacts to ‘actively shape and define disability’ has nonetheless been augmented by insights from STS, as the field emerged concurrently but separately from disability studies in the 1970s.^50^ The material turn in history outlined above was reflected in developments in history of science, in which there had long been an implicit hierarchy of importance between those who worked with tools and those who worked with theories.^51^ Though studies of technology started to become more important in the twentieth century, they were still marked by a tendency towards technological determinism. That is, the tendency to discuss technologies as though they follow their own ‘internal, technical logic that has nothing to do with social relationship’ and the tendency to discuss technological change as causing or determining social change.^52^ In other words, technologies developed because of scientific progress or on their own, and then had corresponding effects on society.^53^ An important corrective to this fallacy arose from scholars within the Edinburgh School of STS, who articulated the social shaping of technology. That is, they emphasised the *social* construction of technology and showed that technological change never happens outside of society.^54^

Against this trend, the idea that ‘technical things have political qualities’—that the stuff and things of technology matter above and beyond social forces—was famously defended by Langdon Winner. For instance, he showed that pneumatic moulding machines were added to a Chicago reaper plant to deskill the workforce and mitigate against the power of the union—the organisers of which were skilled workers.^55^ Similarly, the Shirley colour testing card used in photography to provide the ideal standard for skin colour resulted in the failure of film to capture non-white skin tones.^56^ In these examples, things talk and objects have politics. However, we have been warned not to ‘attribute too much power to the things themselves’ lest we diminish human agency or obscure how material culture has been acted upon.^57^

But objects do have power. The standardised tables used to set thermostats in office buildings mean that office temperatures are consistently too cool for most women.^58^ Airplane cockpits designed to be gender neutral were biased by design against both women and smaller men.^59^ Even standards of weights and designs of machines at certain sizes are political and disabling. As Cynthia Cockburn argues, ‘the appropriation of bodily effectivity on the one hand and the design of machinery and processes on the other have often converged in such a way as to constitute men as capable and women as inadequate’.^60^ Such processes have literally had a disabling impact on women surgeons, who ‘disproportionately report pain and disability from instruments that don't fit their hands’.^61^

Such ‘black boxed’ objects with built-in biases are particularly problematic in the field of healthcare. For instance, Lundy Braun revealed that the data used to set the standards for spirometers (devices used to measure lung capacity) created the ‘scientific fact’ of lower lung capacity in non-white races.^62^ The study of the technological creation of scientific standards thus has clear relevance to how we understand disability. Work by Coreen McGuire has shown that the data sets used to set the standards of normal hearing were artificially elevated by the inter-war telephone system, which increased the total number of people considered to have hearing loss.^63^ The telephone shaped the social relationships of its users as the definitions of different kinds of hearing limitations emerged through this interface between technological development, differential bodily constitutions, and the appropriation of the user's auditory knowledge and values. Disability data gaps embedded in objects have skewed our understanding of the normal.^64^ Sometimes objects, particularly medical objects, do have the power to categorise people and shape their identities.

Theorising objects as agents thus draws attention to the power that the objects we make have in the making of history. Nonetheless, *we make them*. Though both scholars in STS studies and in material culture have emphasised the agency and autonomy of objects, such investment of agency in objects may obscure the agency of the user, which is clearly contrary to the point of disability history to reveal disabled people's agency.^65^ As Ott succinctly explains:

Designating people with disabilities as simply another genre of actants—that is, as equivalent agents with animals, other humans, and inanimate forms—ignores the history of disability and the power wielded by others over “the thing”. Humans, animals, and objects are not equivalent; history and experience demonstrate otherwise. It is true that objects have agency and take on a life of their own, but all actants are not equal, and effect is uneven.^66^

Affordance theory, which postulates that technologies' materiality constrains use to some degree but does not constrain all possible uses, offers ‘a relational middle ground between technological determinism and social constructivism’.^67^ Jaipreet Virdi has developed this more nuanced approach by explaining that we need to understand the positive *and* negative agency of objects to investigate how identity itself can be ‘designed through the material’.^68^ STS and the history of science contain two theoretical strengths that allow us to build on this insight. First, because the idea that users matter was something that had to be established. Second, because the disabled user's embodied knowledge and practiced skill in wielding objects can be considered in parallel to the broad historiography developed around invisible technicians and labour recognised by historians of science as fundamental to the development of scientific knowledge.^69^ In the following two sections, I develop the relevance of these two insights for disability history in turn.

## 3 Section 2: Objects In Use

Roth's work on the Shirley testing card showed how Black *users* of photography developed alternate technological techniques to continue to engage with photography despite its integral limitations. The camera remained in a stage of ‘interpretive flexibility’ as users' innovations with lighting techniques and make-up technologies were actively developed to make the device work for them. This kind of social construction of technology perspective was developed by Weibe Bijker and Trevor Pinch when they argued that different groups of people use the same technologies differently. For instance, the bicycle was used by young male bicycle racers divergently from women and elderly men who wanted a safer vehicle for transport. This pattern of use meant that they gave a new meaning to the high-wheeled bicycle as ‘unsafe’, forcing the development of the safety bicycle.^70^

The insight that *users of technology matter* swelled a new historiographical current at the confluence of the disciplinary streams of history of science and STS. By looking at technology through its users and their usage and by prioritising the mundane aspects of material culture, groups like women were revealed as active consumers of, tinkerers with, and rebels against technology. These groups often used technologies in unanticipated and unexpected ways—the main point of Pinch and Oudshoorn's *How User Matter*, which featured a series of case studies of technologies being used in unforeseen but eventually influential ways. For example, the Model T automobile was used by farmers as a stationery power source, while women in rural areas used the telephone to overcome social isolation in a way that surprised telephone companies.^71^ Users bent technologies to their will.^72^

However, although science and technology (STS) scholars drew our attention to the way that users ‘hack, tinker, consume, and refuse to use technologies to enable a better fit with themselves and their preferences’ they did not consider the importance or indeed the presence of disabled users of technology.^73^ Yet as Bess Williamson has pointed out, ‘disability things often defy the intentions of their makers’.^74^ This historiographical absence was identified in Aimi Hamraie and Kelly Fritsch's 2019 ‘Crip technoscience manifesto’, which further elucidated how knowledge developed by the disabled through object modification has been appropriated without acknowledgement.^75^ Users relationships with objects reveal disabled agency, especially through their modification of devices against the remit of their inventor or designer.

Hamraie and Fritsch's article emphasised that disabled people are experts in technology by highlighting the ‘skills, wisdom, resources, and hacks disabled people utilise for navigating and altering inaccessible worlds’.^76^ They recognised that disability scholars had only engaged in STS scholarship in a limited way, in part because of the aforementioned social model's explicit opposition to medical history, which strongly informs many STS traditions. But more forcefully, they argued that STS have failed to appreciate this kind of use of technology.

However, the point that disabled innovation is an important and recurring phenomenon has been recognised by disability historians.^77^ The way that disabled innovation has been commodified was the explicit focus of Claire Jones' 2017 edited collection on prostheses and commodity cultures.^78^ Bess Williamson's work extended such analysis to reveal the importance of dyad disability invention by revealing the origin of the OXO grip line of kitchen tools' invention by a husband and wife—whose expertise was appropriated and publicly erased.^79^ Individual personalities and preferences have been central to Jaipreet Virdi's material analysis of disability artefacts which were explicitly ‘modified to forge personal identities—of personality, preference, and even medical diagnosis’. In this work, ‘people with disabilities are cast not just as users, but as agents of technological change’.^80^ Although much of this work focuses on everyday and domestic technologies, there is growing consensus that disabled innovation has long been central to science (which was largely domestic until the modern period).^81^

## 4 Section 3: Objects As Science

Disability historians who work on the history of technologies have started to argue for the importance of investigating the entirety of disabled people's lives, including their use and adaptation of medical objects and scientific theories.^82^ Proponents of the design model emphasise that ‘disabled people themselves also used design to adapt or invent their own devices and, in some cases, transform industries’.^83^ Blind engineer James Swail designed electronic devices explicitly for blind people working in science, technology and engineering.^84^ Less directly, the way talking books were used by blind users prompted important innovations in music and voice recognition technologies by divorcing frequency from speed.^85^ As Mara Mills has shown, the expertise of deaf and deaf-blind inventors was crucial to the development of speech theory and auditory equipment within the telephone company AT&T.^86^ Everyday functional objects of disability are thus ‘embodiments of practices’.^87^

When it comes to disability history, technologies are not only representations of experience but also materialised forms of knowledge.^88^ This means that when historians attend to materials, we can illuminate the scientific contributions of marginalised traditions of knowledge-making, including indigenous knowledge and disabled innovation.^89^ STS scholars have long considered technologies to comprise ‘the objectification of knowledge and practices in new material forms’ and this insight can support the move of disability history into scientific spaces.^90^

The tacit knowledge and embodied knowledge fundamental to disabled innovation is reinforced by work in the history of science that has redefined making as knowing and knowledge as practice. Disabled users' embodied knowledge and practiced skill can thus be considered as legitimately scientific in the same way that early modern artisanal knowledge has been recognised by historians like Pamela Smith as fundamental to the formation of the new scientific knowledge.^91^ Household based ‘thrifty’ scientific practices that involved the creative refashioning of everyday objects were integral to shaping scientific epistemologies and methodologies.^92^ In addition, disabled users' contributions have often been collaborative, sometimes out of necessity, sometimes because of an increased tendency to avoid patenting practices and support the altruistic movement of such knowledge.^93^

Criticisms of the tendency to neglect collaborative work in science was fundamental to Steven Shapin's 1989 work on invisible technicians, which concluded that invisible technicians were made invisible because of western culture's bias towards individual rather than collective work.^94^ Shapin's article led to a plethora of works that deepened our understanding of what activities constituted science, revealed other scientific actors, and demonstrated that different ways of knowing were considered as valid depending on particular historical circumstances. The role of ‘invisible technicians’ has been highlighted as particularly important to group work processes, in which ‘the tension between formal task descriptions and overt work on the one hand, and informal tasks and “behind the scenes” work on the other, has been an important consideration’.^95^ This is clearly relevant to the work uncovering the inventions of disabled dyads and families, which can usefully be linked to research that has valued ‘the collective knowledge of a family’ rather than individual endeavour.^96^ Disabled innovation could take place within the context of a community, such as those reliant on respirators in the twentieth century, whose collaborative adaptations of household technologies demonstrate how ‘Disabled users situated knowledge—their mulling, imagining, articulating—was collectively mobilised into design solutions and indeed, to scientific knowledge’.^97^

## 5 Section 4: Objects As Subjects

Disability history has placed a particular imperative on recovering lived experience and hidden histories. Analysis of material objects is one way of revealing such hidden histories. But this method tends to support histories of disabilities that are and were visible; it is limited when it comes to disability that does not so clearly connect with materiality. Therefore, despite the conceptual difficulties associated with integrating disability history and medical history, medical history and indeed, history of psychiatry, does offer strategies of relevance to the disability historian.

For example, historians of psychiatry have used semi-verbatim stenographic case note transcripts to directly recover patient voices and show how patient stories were collaboratively created: ‘These materials, while infused with the authorial agency of their psychiatrists, unveil a history of patient–psychiatrist dialogues in which the power to define the “story of illness” ebbs and flows between both parties’.^98^ For others, patient voices have been more directly sought through oral histories that highlight patient agency and explore their role as narrators by excavating ‘the spaces in which people tell their stories—the ways in which they negotiate language, metaphor, and presentation of self’.^99^ More literal spaces are central to the work of Sarah Holland, who has explored how asylum patient engagement with, experiences of, and refusal to do farm work can reveal patient identities in flux between the spaces of the hospital and the outside world.^100^ In this work actions speak louder than words and innovative methodologies focused on movement allow us to recover the patient's view indirectly.

Holland's work was published in Anne Hanley and Jessica Meyer's 2021 edited collection on patient voices, which also contained an important call from Michael Worboys for medical historians to shift their attention from patients to the actions of non-patients to highlight the numerous ways in which people care for themselves and make health choices outside of the medical sphere.^101^ His argument made a key methodological point in support of this shift, arguing that ‘historians have tended to study the diseases that medicine prioritises. Histories are biased in favour of mortality over morbidity, acute over chronic disease, and serious over slight complaints. With each of these pairs, the latter was and is overwhelmingly the lived experience of illness and disease’.^102^ Another way of putting this would be to say that medical historians ought to be doing disability history. Although it is critical not to simply equate disease with disability, Linker and Abel have argued that ‘A disability approach transforms traditional disease histories by expanding them to include actors outside the clinic and larger political issues such as social welfare, citizenship, and belonging’.^103^

These are issues that are essential to work within critical medical humanities, which have long prioritised the importance of lived experience alongside (and over) the limitations of biomedical knowledge. In 2015, Viney, Callard, and Woods asked, ‘Can the medical humanities intervene more explicitly in *ontological* questions—in particular, of aetiology, pathogenesis, intervention and cure—rather than, as has commonly been the case, leaving such questions largely to the domains of the life sciences and biomedicine?’^104^

Historians of science's focus on science's epistemological power can allow disability historians to question the creation of disability *as a ‘scientific fact’* and so offers one way to answer these kinds of questions.^105^ For example, the nineteenth-century development of the ophthalmoscope standardised visual acuity in relation to a numerical measure of ‘normal’ vision related to an arbitrary standard of efficiency: ‘Vision testing by test-types standardised the “normal” visual acuity of humans so as to determine the degree of a person's refractive anomaly but it was not an average that fully reflected the population; it could be succeeded or exceeded’.^106^ Scholarship from history of science on measurement, statistics, and classification systems rests on the premise that science itself *is social* in a way that the social/medical model binary can obscure.^107^ These methodologies enable us to criticise the statistical construction of normalcy which disablement is defined against.^108^

Penny Richards and Susan Burch have argued that administrative and institutional data do not ‘tell the stories that disability scholars seek most to retrieve’.^109^ They argue that such data sets cannot tell us anything about the lived experience of disability and will reinforce ableist assumptions. While it might be true that lived experience is less accessible in such historical sources, I contend that these kinds of sources can be extremely valuable for revealing the scientific and statistical construction of disability. In the case of medical devices, we often find, for instance, that ‘their most unique and significant characteristics can be found in the algorithms used to program them, rather than in their physical manifestation’.^110^ However, these possibilities have not yet been realised, despite their obvious potential. Mara Mills, Jaipreet Virdi, and Sarah F. Rose explain in their call for a disability history of science, ‘Similar to the theories of medicalisation and the “social construction of *x*,” historical epistemology has revealed specific disabilities to be contingent on the cultural order, without questioning the broader category of disability itself’.^111^

Disability is constructed by design and shaped by the environment—but at the causal level it is constructed by science. Matter matters, but statistics count. Marion Schmidt's recent work on genetic deafness is an exemplar of the effectiveness of a history of science approach to disability history.^112^ Working out how and when disability has been established as an ‘objective’ departure from ‘normal functioning’ widens the lens of the disability historian. The scientific categorisation of disability as natural and inevitable can only be uncovered by critically questioning the legitimacy of the paradigms that support its framing. When Peter Dear posed the question of what history of science was the history *of* he concluded that we must acknowledge ‘that science is not one thing, a natural kind, while at the same time recognising that the symbol “science” is culturally very real indeed’.^113^ This dual understanding can allow disability historians to investigate and to reframe disabled innovation as properly part of science and simultaneously destabilise its power by uncovering how scientific processes have defined and hidden disability in history.

## 6 Conclusion

The difficulty of using methodologies from STS and history of science to understand disabled adaptation of medical objects not only reproduces the social/medical model binary; it reflects the long tradition of distinguishing between science/mind/theory in opposition to art/bodies/practice. In 1995, John Harley Warner argued that boundaries between history of science and history of medicine were artificially perpetuated and misused by historians as a way to ‘organise the past conceptually’ which was essentially ‘rooted in sibling jealousy and the quest for separate identities by fields that long shared their marginality to the historical mainstream’.^114^ Devaluing and decrying the value of a particular field is an important way in which new disciplines work to achieve legitimacy and professionalisation. Warner argued that the new social histories of the 1970s developed within the mainstream of history and therefore were not writing in relation to the freighted historiographies of history of science and medical history that had developed in the preceding 40 years. Because they had not developed out of a defensive relationship this meant they were using different methodologies, using different sources, and asking different questions and this different focus was (and arguably still is) characterised by historians working in history of science and medical history as lacking rigour. On the other hand, social historians set up traditional medical history as inherently whiggish and anachronistic so they could rally around a new historiography defined against it.

However, disability historians have a responsibility to the present as well as the past and the insistence within both history of science and medical history that we avoid considering how the problems of modern biomedicine relate to the past lest we commit the sin of anachronism does not mesh well with this responsibility. ‘Nothing about us without us’ is more than a mantra and understanding the impact of materials and the material circumstances that shape disabled lives remains fundamental to guarding the future of the subject.

Turning to objects reveals that the boundaries between STS and medical history and disability history are fluid. Objects are devices that build bridges between disciplines, allowing us to move away from manufactured discipline demarcation and towards the inextricable combination of the scientific and the personal, collapsing dichotomies between mind/body and subject/object.

**Figure 1:**
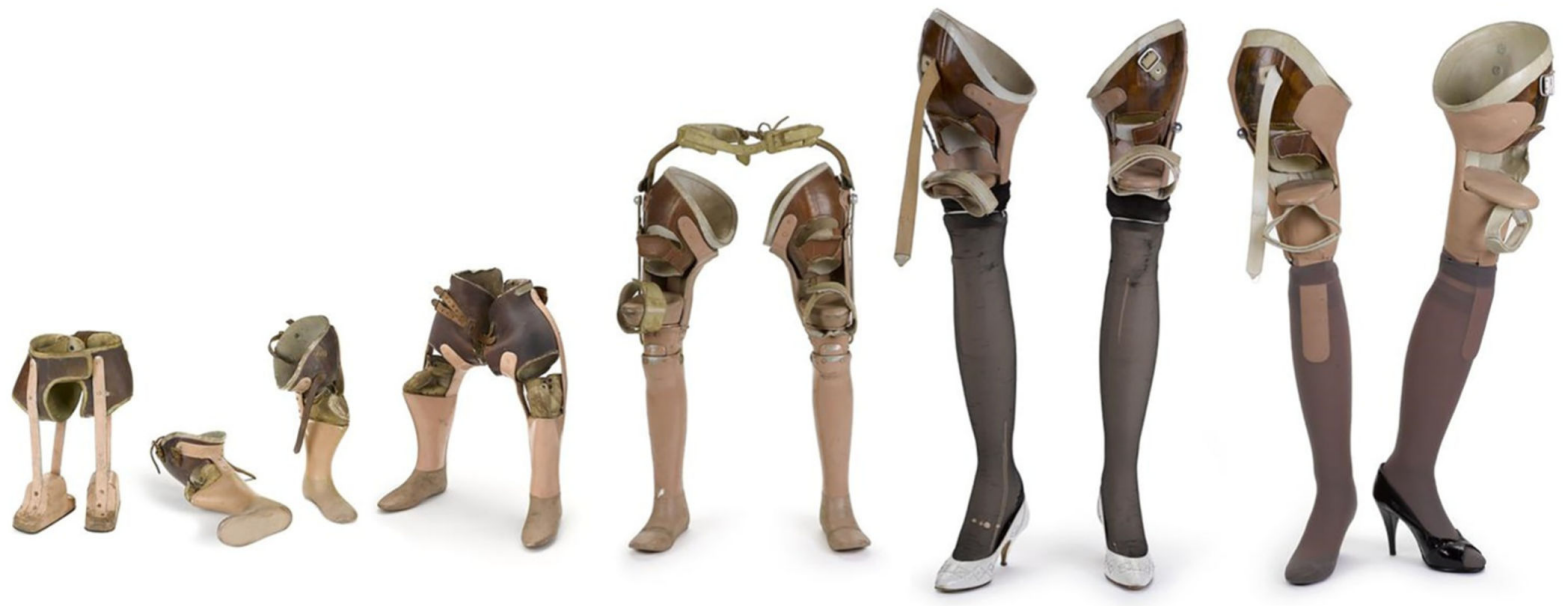
Shoevolution. Tracey Baynam's prosthetic limbs. Image released under a Creative Commons Attribution-NonCommercial-ShareAlike 4.0 Licence.

Consider, for instance, the prosthetic/artificial limbs with attached four-inch stilettos used by Tracey Baynam and held in the Science Museum (Figure 1, far right).

These heels demonstrate Tracey's agency, as ‘she persuaded her limb fitter Fred to make’ them, something he did voluntarily.^115^ His co-operation further demonstrates a more complex and ambivalent relationship between prosthetic users and the ‘medical establishment’ (here represented by Fred) showing collaborative invention and making together between user and designer. In addition, there is a tendency to think more about technologies that are considered to have driven innovation or marked the start of historical epochs, and less about the technologies (the motorways, the concrete, the cups of tea, the electric grid) that surround us and undergird our existence. Similarly, that technologies are often coded as masculine elides the fact that clothes, make-up, handbags, and shoes are powerful technologies, allowing us to move in the world, into previously inaccessible spaces, while literally moulding our bodies; ‘As much as the foot shapes the shoe, the shoe shapes the foot’.^116^ As Hilary Davidson shows, shoes are empathetic and emotional vessels, and this is strongly articulated in Susan Ward's moving remembrance of when, in a moment of role-reversal, she bought her mother her first pair of ‘real shoes’ when she moved out of braces and into a wheelchair.^117^ Tracey's heeled shoes are powerful, sexual objects, walking a line between stigma, sexuality and respectability especially difficult for disabled women to traverse.^118^ They point the way to a new methodological agenda for disability history that integrates insights from history of science and STS. They are vessels that contain the messy stuff that disability history must hold within it.

## End Notes

1 Some theorists have placed the social model at the end of a chronological progressive track that purportedly moved from a moral model to a medical model, but this simplistic approach has not gained much purchase in history. For a cogent critique see Aimi Hamraie, ‘Historical Epistemology as Disability Studies Methodology: From the Models Framework to Foucault's Archaeology of Cure’ *Foucault Studies* 19 (2015) b.

2 On postmodernism as used by historians see Callum Brown, *Postmodernism for Historians* (London: Pearson, 2005); on material culture see Selena Dyer, ‘State of the Field: Material Culture’ *History* 106:370 (2021) p. 283.

3 Ludmilla Jordanova, *History in Practice*, 3rd edition (Bloomsbury Academic: London and New York, 2020) p. 128.

4 Penny Richards and Susan Burch, ‘Documents, Ethics, and the Disability Historian’ in *The Oxford Handbook of Disability History* edited by M. Remis, C. Kudlick and K. E. Nielsen (Oxford, Oxford University Press, 2018) 161–174 (p. 162).

5 Ludmilla Jordanova, *History in Practice*, 3rd edition (Bloomsbury Academic: London and New York, 2020) p. 128.

6 Deborah Stone, *The Disabled State* (Basingstoke: Macmillan, 1985); Anne Borsay, *Disability and Social Policy in Britain since 1750: a History of Exclusion* (Basingstoke: Palgrave Macmillan, 2005); Deborah Cohen, *The War Come Home: Disabled Veterans in Britain and Germany, 1914–1939* (Berkeley: University of California Press, 2001); Julie Anderson, *War, Disability and Rehabilitation in Britain: ‘Soul of a Nation’* (Manchester, Manchester University Press, 2011); Douglas C. Baynton, *Defectives in the Land: Disability and Immigration in the Age of Eugenics* (Chicago: University of Chicago Press, 2016). For an excellent review of the state of the field see Daniel Blackie and Alexia Moncrieff, ‘State of the Field: Disability History’ *History* 107:377 (2022) 1–23 https://doi.org/10.1111/1468-229X.13315.

7 Penny Richards and Susan Burch, ‘Documents, Ethics, and the Disability Historian’ in *The Oxford Handbook of Disability History* edited by M. Remis, C. Kudlick and K. E. Nielsen (Oxford, Oxford University Press, 2018) 161–174.

8 The project website with associated resources and publications is maintained here https://www.dis-ind-soc.org.uk/en/publications.htm.

9 David M. Turner and Daniel Blackie, *Disability in the Industrial Revolution: Physical Impairment in British Coalmining, 1780–1880* (Manchester: Manchester University Press, 2018); Daniel Blackie, ‘Disability and Work During the Industrial Revolution in Britain’, in M. Rembis, C. Kudlick, and K. E. Nielsen (eds.), *The Oxford Handbook of Disability History* (Oxford: Oxford University Press).

10 Vic Finklestein, ‘Disability and the helper/helped relationship. An historical view’ in *Handicap in a Social World* edited by A. Brechin, P. Liddiard and J. Swain (London: Hodder and Stoughton, 1981).

11 Jessica Meyer, ‘“Not Septimus Now”: wives of disabled veterans and cultural memory of the First World War in Britain’ *Women's History Review* 13:1 (2004) is an exemplar of this work; See also Kirsti Bohata, Alexandra Jones, Mike Mantin, and Steven Thompson, *Disability in Industrial Britain: A Cultural and Literary History of Impairment in the Coal Industry, 1880–1948* (Manchester, Manchester University Press); Alexandra Jones, “‘Her body [was] like a hard-worked machine’: Women's Work and Disability in Coalfields Literature, 1880–1950,” *Disability Studies Quarterly* 37:4 (2017).

12 Ann Laura Stoler, *Along the Archival Grain: Epistemic Anxieties and Colonial Common Sense*. (New Jersey: Princeton University Press, 2008).

13 Penny Richards and Susan Burch, ‘Documents, Ethics, and the Disability Historian’ in *The Oxford Handbook of Disability History* edited by M. Remis, C. Kudlick and K. E. Nielsen (Oxford, Oxford University Press, 2018) 161–174 (p. 164. and p. 166.).

14 For these early feminist perspectives see Jenny Morris, ‘Personal and political; A Feminist Perspective on Researching Physical Disability’ *Disability Handicap Soc* 7:2 (1992) 157–166 and Susan Wendell, *The Rejected Body: Feminist Philosophical Reflections on Disability* (London: Routledge); for its integration with queer theory see Alison Kafer, ‘Compulsory Bodies: Reflections on Heterosexuality and Able-Bodiedness’ *Journal of Women's History* 15:3 (2003) 77–89; for this point in relation to pain see Tobin Siebers, ‘Disability in Theory: From Social Constructionism to the New Realism of the Body’ *American Literary History* 13:4 (2001) 737–754; for the value-neutral model as an alternative see Elizabeth Barnes, *The Minority Body: a Theory of Disability* (Oxford: Oxford University Press, 2016).

15 Elizabeth Guffey and Bess Williamson, ‘Introduction: Rethinking Design History through Disability, Rethinking Disability through Design’ in *Making Disability Modern: Design Histories* edited by Elizabeth Guffey and Bess Williamson (London, Bloomsbury, 2020) 1–14, p. 6.

16 And in part because of the disciplinary separation between history of science and history of medicine.

17 Beth Linker, ‘On the Borderland of Medical and Disability History: A Survey of the Fields’ *Bulletin of the History of Medicine* 87:4 (2013) 499–535 (p. 502).

18 Elizabeth Guffey and Bess Williamson, ‘Introduction: Rethinking Design History through Disability, Rethinking Disability through Design’ in *Making Disability Modern: Design Histories* edited by Elizabeth Guffey and Bess Williamson (London, Bloomsbury, 2020) 1–14, p. 4.

19 On the pioneering work of Deaf historians see Catherine Kudlick, ‘Disability History: Why We Need Another Other’ *The American Historical Review* 108:3 (2003).

20 For the argument that Deaf people should not label themselves as disabled see Harlan Lane, ‘Do Deaf People Have a Disability?’ *Sign Language Studies* 2:4 (2002) 356–379 (pp. 368–369).

21 This positioning of disability as an analytic category in this way appeared in Paul K. Longmore and Lauri Umanski, *The New Disability History: American Perspectives* (New York: New York University Press, 2001) The idea of disability as ‘another other’ was expressed in Catherine Kudlick, ‘Disability History: Why We Need Another Other’ *The American Historical Review* 108:3 (2003); for examples of this work see Esme Cleall, ‘Colonising Disability: Impairment and Otherness Across Britain and Its Empire’, c. 1800–1914 (Cambridge: Cambridge University Press, 2022).

22 Michael Rembis, Catherine Kudlick, Kim E. Nielsen, ‘Introduction’ to *The Oxford Handbook of Disability History* (Oxford Handbooks, Oxford Academic, 2018) p. 9.

23 Andrew Hogan, ‘Moving away from the “Medical Model”: The Development and Revision of the World Health Organization's Classification of Disability’ *Bulletin of the History of Medicine* 93:2 (2019) 241–269.

24 See for instance early feminist medical history like Ludmilla Jordanova “Gender, generation and science: William Hunter's obstetrical atlas.” In *William Hunter and the Eighteenth-Century Medical World*, edited by W.F. Bynum and Roy Porter, 385–412. Cambridge: Cambridge University Press, 1983 or Lisa Forman Cody, “The Politics of Reproduction: From Midwives' Alternative Public Sphere to the Public Spectacle of Man-Midwifery.” *Eighteenth-Century Studies*, 32:4 (1999): 477–495.

25 Flurin Condrau, ‘The Patient's View Meets the Clinical Gaze’ Social History of Medicine 20:3 (2007) 525–540 (p. 528).

26 Robert Chapman, ‘Neurodiversity, disability, wellbeing’; in *Neurodiversity studies: A New Critical Paradigm* edited by Hanna Rosqvist, Nick Chown, and Anna Stenning (Routledge, Abingdon, 2020) p. 63. Shelley Tremain has also argued that we need to attend to the historical forces that have materialised impairments as natural, See Shelley Tremain, ‘On the Government of Disability’ *Social Theory and Practice* 27:4 (2001).

27 Aimi Hamraie, ‘Historical Epistemology as Disability Studies Methodology: From the Models Framework to Foucault's Archaeology of Cure’ *Foucault Studies* 19 (2015) 108–134. P. 110 and p. 122.

28 John Haley Warner, “The History of Science and the Sciences of Medicine,” *Osiris* 10 (1995): 164–193, p. 175. See this piece for more background on how and why these disciplines developed differently.

29 John Haley Warner, “The History of Science and the Sciences of Medicine,” *Osiris* 10 (1995): 164–193, p. 175.

30 Katherine Ott and David Serlin, *Artificial Parts, Practical Lives: Modern Histories of Prosthetics* (New York University Press, 2002).

31 Katherine Ott, ‘Disability Things: Material Culture and American Disability History, 1700–2010’ in *Disability Histories* edited by S. Burch and M. Rembis (University of Illinois Press, 2014) p. 120.

32 Katherine Ott and David Serlin, *Artificial Parts, Practical Lives: Modern Histories of Prosthetics* (New York University Press, 2002) p. 4.

33 Raman Srinivasan, ‘Technology Sits Cross-Legged: Developing the Jaipur Foot Prosthesis’ in K. Ott and D. Serlin, *Artificial Parts, Practical Lives: Modern Histories of Prosthetics* (New York University Press, 2002) p. 337.

34 Elizabeth Guffey and Bess Williamson, ‘Introduction: Rethinking Design History through Disability, Rethinking Disability through Design’ in *Making Disability Modern: Design Histories* edited by E. Guffey and B. Williamson (London, Bloomsbury, 2020) 1–14, p. 5.

35 Debby Sneed, ‘The architecture of access: ramps at ancient Greek healing sanctuaries’ *Antiquity* 94: 376 (2020) 1015–1029, p. 1022.

36 Bess Williamson, *Accessible America*: *A History of Disability and Design* (New York University Press, 2019) p. 5.

37 Gemma Almond, ‘Why the anonymous and everyday objects are important: using the Science Museum's collections to re-write the history of vision aids’ *Science Museum Group Journa*l 13 (2020) https://doi.org/10.15180/201306/001.

38 Katherine Ott, ‘Disability Things: Material Culture and American Disability History, 1700–2010’ in *Disability Histories* edited by S. Burch and M. Rembis (University of Illinois Press, 2014) p. 120 and p. 122. See also, Nicole Belolan, ‘The Material Culture of Gout in Early America’ in E. Guffey & B. Williamson (Eds.), *Making Disability Modern: Design Histories* (London: Bloomsbury, 2020) pp. 19–42.

39 Woods, Brian, and Nick Watson, ‘In Pursuit of Standardization: The British Ministry of Health's Model 8F Wheelchair, 1948–1962’ *Technology and Culture* 45:3 (2004) 540–568.

40 John Gagne, ‘Emotional Attachments: Iron Hands, their Makers, and their Wearers, 1450–1600’ in Stephanie Downes, Sally Holloway and Sarah Randles, *Feeling Things: Objects and Emotions through History* (Oxford, Oxford University Press, 2018) (133–153) p. 134.

41 Katherine Ott, ‘Disability Things: Material Culture and American Disability History, 1700–2010’ in Di*sability Histories* edited by S. Burch and M. Rembis (Illinois: University of Illinois Press, 2014) p. 120.

42 It was only when physically picking up an early integrated telephone that I realised the transistor and receiver were designed to adjust to an individual's face size and shape, ensuring that one's mouth and ear were directly placed within them. This materially evidenced how hard the early telephone was to hear.

43 Coreen McGuire and Havi Carel, ‘The Visible and the Invisible: Disability, Assistive Technology, and Stigma’, in A. Cureton, and D. T. Wasserman (eds.), *The Oxford Handbook of Philosophy and Disability* (online edn, Oxford; Oxford University Press, 2018), p. 602, See also Graeme Gooday and Karen Sayer, *Managing the Experience of Hearing Loss in Britain, 1830–1930*. (London: Palgrave Macmillian, 2017); Cathy Sarli et al., ‘19th-Century Camouflaged Mechanical Hearing Devices’ *Otology & Neurotology*, 24:4 (2003) 691–698.

44 Gemma Almond-Brown, *Spectacles and the Victorians: Measuring, defining and shaping visual capacity* (Manchester, Manchester University Press, 2023) p. 23.

45 Stephanie Downes, Sally Holloway and Sarah Randles, *Feeling Things: Objects and Emotions through History* (Oxford, Oxford University Press, 2018) p. 2.

46 Sally Holloway, ‘Materializing Maternal Emotions: Birth, Celebration, and Renunciation in England, c.1688–1830’ in Stephanie Downes, Sally Holloway and Sarah Randles, *Feeling Things: Objects and Emotions through History* (Oxford, Oxford University Press, 2018) 154–172.

47 Jaipreet Virdi, ‘Brett and Toby: Asserting the Disabled Gaze’ *Winterthur Portfolio* 57:1 (2023).

48 These kinds of object histories can also be recovered through the development of appropriately detailed digital resources; for instance, see Homepage · Objects of Disability (omeka.net).

49 Karen Harvey, *History and Material Culture* (Abingdon: Routledge, 2016) p. 6.

50 Katherine Ott, ‘Disability Things: Material Culture and American Disability History, 1700–2010’ in *Disability Histories* edited by Susan Burch and Michael Rembis (Illinois: University of Illinois Press, 2014) p. 119.

51 Eric Schatzberg, ‘Technik Comes to America’ *Technology and Culture* 47: 3 (2006) 486–512 (p. 489).

52 Sally Wyatt, ‘Technological determinism is dead, long live technological determinism’ in *The Handbook of Science and Technology Studies*, Third Edition, edited by Edward J. Hackett, Olga Amsterdamska, Michael Lynch, Judy Wajcman (Cambridge, MA: MIT Press, 22007).

53 Donald Mackenzie and Judy Wajcman ‘Introduction’ to *The Social Shaping of Technology* edited by Donald Mackenzie and Judy Wajcman (Buckingham: Open University Press, 1999).

54 Donald Mackenzie and Judy Wajcman ‘Introduction’ to *The Social Shaping of Technology* edited by Donald Mackenzie and Judy Wajcman (Buckingham: Open University Press, 1999) p. 8/50.

55 Langdon Winner, *The Whale and the Reactor: A Search for Limits in an Age of High Technology* (Chicago: University of Chicago Press, 1989) p. 24. Winner's more famous 1980 article in *Daedalus* on the racist impacts of the low overpasses on Long Island has become ambivalent, characterised in 1999 as an ‘urban legend in S&TS’ by Steve Woolgar and Geoff Cooper in ‘Do Artefacts Have Ambivalence? Moses’ Bridges, Winner's Bridges and Other Urban Legends in S&TS’ *Social Studies of Science* 29:3 (1999) 433–448.

56 Lorna Roth, ‘Making Skin Visible through Liberatory Design’ in Ruha Benjamin *Captivating Technology: Race, Carceral Technoscience, and Liberatory Imagination in Everyday Life* (New York: Duke University Press, 2019) See also Richard Dyer, ‘Lighting for Whiteness’ in *The Light of the World* (Routledge: London and New York, 1997).

57 Samuel J. M. M. Alberti, ‘Objects and the Museum’ *Isis* 96:4 (2005) P. 561.

58 Joosy van Hoof. ‘Female thermal demand’. *Nature Climate Change* 5, (2015) 1029–1030. https://doi.org/10.1038/nclimate2755.

59 Rachel N. Weber, ‘Manufacturing Gender in Commercial and Military Cockpit Design’ *Science, Technology and Human Values* 22:2 (1997) 139–264.

60 Cynthia Cockburn, ‘The Material of Male Power’ *Feminist Review* 9 (1981) 41–58 (p. 51).

61 Shen-yi Liao and Vanessa Carbonell, ‘Materialised Oppression in Medical Tools and Technologies’ *The American Journal of Bioethics* 23:4 (2022) 9–23.

62 Lundy Braun, *Breathing Race into the Machine: The Surprising Career of the Spirometer from Plantation to Genetics* (University of Minnesota Press: Minneapolis, 2014).

63 Coreen McGuire, ‘The categorisation of hearing loss through telephony in inter-war Britain’ *History and Technology* 35:2 (2019); for the American context see Mara Mills, ‘Deafening: Noise and the Engineering of Communication in the Telephone System’ *Grey Room* 43:7 (2011) 118–143.

64 Alice Malpass, Coreen McGuire, Jane Macnaughton ‘The body says it’: the difficulty of measuring and communicating sensations of breathlessness. *Medical Humanities* 48:1 (2022) 63–75.

65 Selena Dyer, ‘State of the Field: Material Culture’ *History* 106:370 (2021) p. 285.

66 Katherine Ott, ‘Disability Things: Material Culture and American Disability History, 1700–2010’ in *Disability Histories* edited by Susan Burch and Michael Rembis (University of Illinois Press, 2014) p. 124.

67 Huifen Wang, Jialu Wang, Qiuhong Tang, ‘A Review of Application of Affordance Theory in Information Systems’ *Journal of Service Science and Management* 11 (2018) 56–70 (p. 57). For the original articulation of affordance theory in relation to postphenomenology see Don Ihde, T*echnology and the Lifeworld: From Garden to Earth* (Indiana University Press, 1990).

68 Jaipreet Virdi, ‘Materializing User Identities and Digital Humanities’ in *Making Disability Modern: Design Histories* edited by Elizabeth Guffey and Bess Williamson (London, Bloomsbury, 2020) 225–251. p. 229.

69 Jenny Bangham, Xan Chacko, Judith Kaplan, Invisible Labour in Modern Science (London and Maryland: Rowman and Littlefield); Pamela H. Smith, Amy R. W. Meyers and Harold J. Cook (eds.) *Ways of Making and Knowing*; The Material Culture of Empirical Knowledge (Ann Arbor: University of Michigan Press, 2014); Pamela H. Smith and Tonny Beentjes, ‘Nature and Art, Making and Knowing: Reconstructing Sixteenth-Century Life-Casting Techniques’ Renaissance Quarterly 63:1 (2010) 128–179 (p. 168 and 175).

70 Trevor J. Pinch and Wiebe E. Bijker, ‘The Social Construction of Facts and Artefacts: Or How the Sociology of Science and the Sociology of Technology Might Benefit Each Other’ *Social Studies of Science* 14:3 (1984) 399–441.

71 Nelly. E. J. Oudshoorn and Trevor J. Pinch, *How users matter: The co-construction of users and technologies* (Cambridge, Massachusetts: The MIT Press, 2003) For gender and the telephone see also, Claude S Fischer, ‘Gender and the residential telephone, 1890–1940: Technologies of sociability’ 3:2 *Sociological Forum* (1998) 211–233.

72 For more examples of this historiography see Ruth Oldenziel and Mikael Hård, *Consumers, Tinkerers, Rebels: The People who Shaped Europe* (New York: Palgrave Macmillan, 2013); Eric Von Hippel, ‘The dominant role of users in the scientific instrument innovation process’ *Research policy* 5:3 (1976) 212–239 Sally Wyatt, ‘Non-users also matter: The construction of users and non-users of the Internet’ in N. E. J. Oudshoorn and T. Pinch, *How users matter: The co-construction of users and technologies* (Cambridge, Massachusetts: The MIT Press, 2003).

73 Coreen McGuire, ‘Who Invented the Possum? What Historians Can Learn from Disabled Innovation in Britain's Responaut Communities’ *Technology and Culture* 65:1 (2024): 89–116 (p. 90.). See also, Stuart Blume, Vasilis Galis, and Andrés Valderrama Pineda (guest eds.), “STS and Disability” Special Issue of *Science, Technology & Human Values*, 39.1 (2014).

74 Bess Williamson, *Accessible America: A History of Disability and Design* (New York: New York University Press, 2019) p. 9.

75 Aimi Hamraie and Kelly Fritsch ‘Crip technoscience Manifesto’ *Catalyst* 5:1 (2019) Such commodification was the subject of Claire Jones' edited collection on prostheses in commodity cultures, see Claire L. Jones (ed.) *Rethinking modern prosthesis in Anglo-American commodity cultures, 1820–1939* (Manchester, Manchester University Press, 2017).

76 A Hamraie and K Fritsch ‘Crip technoscience Manifesto’ *Catalyst* 5:1 (2019) p. 3. Cassandra Hartblay has extended Hamraie and Fritsch's discussion of disabled expertise in design to articulate a much broader kind of disabled expertise to emphasise its role in managing relationships and relationality, see C. Hartblay, ‘Disability expertise: claiming disability anthropology’ *Current Anthropology* 61: S21 (2020).

77 Emily Rapp, ‘Beautiful Monsters: Life with a Prosthetic Limb’ in J. Dyer, J. Cognard-Black, & E. McLeod Walls (Eds.), *From Curlers to Chainsaws: Women and Their Machines* (University of Michigan Press, 2016); J. S. Reznick, ‘Prostheses and Propaganda: Materiality and the Human Body in the Great War’ in N. Saunders, Matters of Conflict: Material Culture, Memory and the First World War (Routledge: Abingdon, 2004) (pp. 51–62). Routledge; Coreen McGuire, *Measuring Difference, Numbering Normal: Setting the standards for disability in the interwar period* (Manchester, Manchester University Press, 2020).

78 Such commodification was the explicit focus of Jones, Clare, L., ed. *Rethinking modern prosthesis in Anglo-American commodity cultures, 1820–1939* (Manchester: Manchester University Press, 2017).

79 Bess Williamson “Getting a Grip: Disability in American Industrial Design.” *Winterthur Portfolio*, 46:4 (2012): 213–236.

80 Jaipreet Virdi, ‘Materializing User Identities and Digital Humanities’ in *Making Disability Modern: Design Histories* edited by Elizabeth Guffey and Bess Williamson (London, Bloomsbury, 2020) 225–251. p. 229.

81 Famous deaf inventors like Thomas Edison, Oliver Heaviside, and John Ambrose Fleming have been given most attention, for example, see, Harry G. Lang and Bonnie Meath-Lang, “Guillaume Amontons,” in *Deaf Persons in the Arts and Sciences: A Biographical Dictionary*, eds. Harry G. Lang and Bonnie Meath-Lang (Westport, CT: Greenwood Press, 1995); This focus might be because Deaf history emerged as the first and strongest component of disability history, though it this is sometimes consciously articulated as separate from disability. The minor inventions of non-famous deaf users of telephony are catalogued in Coreen McGuire, *Measuring Difference Numbering Normal: Setting the standards for disability in the interwar period* (Manchester: Manchester University Press, 2020).

82 For an early articulation of this need see, Julie Anderson, *War, Disability and Rehabilitation in Britain: ‘Soul of a Nation’* (Manchester, Manchester University Press, 2011); Julie Anderson and Neil Pemberton, ‘Walking Alone: Aiding the War and Civilian Blind in the Inter-war Period’ European Review of History 14:4 (2007) 459–479 and for more recent book-length examples of investigations of technology from a disability history perspective see Jaipreet Virdi, *Hearing Happiness: Deafness Cures in History* (Chicago: University of Chicago Press, 2020); Graeme Gooday and Karen Sayer, *Managing the Experience of Hearing Loss in Britain, 1830–1930*. (London: Palgrave Macmillian, 2017); Coreen McGuire, *Measuring Difference, Numbering Normal: Setting the standards for disability in the interwar period* (Manchester, Manchester University Press, 2020).

83 Elizabeth Guffey and Bess Williamson, ‘Introduction: Rethinking Design History through Disability, Rethinking Disability through Design’ in *Making Disability Modern: Design Histories* edited by Elizabeth Guffey and Bess Williamson (London, Bloomsbury, 2020) 1–14, p. 3.

84 Beth A. Robertson, ‘“Rehabilitation aids for the blind”: disability and technological knowledge in Canada, 1947–1985’ *History and Technology* 36:1 (2020) 30–53 (p. 33).

85 Matthew Rubery, Mills, M. (2013). Print Disability: The Co-Construction of Blindness and Reading. In U. Bergermann (Ed.), *Disability Trouble* (pp. 195–204). b_books. Mills, M. (2015). Technology. In R. Adams, B. Reiss & D. Serlin (Eds.) in Rachel Adams, Benjamin Reiss, and David Serlin (eds.), *Keywords for Disability Studie*s (pp. 176–179). New York University Press.

86 Mara Mills, ‘On Disability and Cybernetics: Helen Keller, Norbert Wiener, and the Hearing Glove’ *Differences: A Journal of Feminist Cultural Studies* 22:2–3 (2011) 74–111 (p. 85).

87 Elizabeth Newswald, ‘Things That Don't Talk Much and Things That Feel: Developing a Material Culture Methodology for “Black Box” Medical Devices’ *Nuncius* 35:3 (2020) 632–659 (p. 644).

88 Coreen McGuire, ‘Who Invented the Possum? What Historians Can Learn from Disabled Innovation in Britain's Responaut Communities’ *Technology and Culture* 65:1 (2024): 89–116 (p. 90).

89 Helen Tilley, ‘Global Histories, Vernacular Science, and African Genealogies; or, Is the History of Science Ready for the World?’ *Isis* 101 (2010) 110–119.

90 Lucy Suchman, ‘Working Relations of Technology Production and Use’ *Computer Supported Cooperative Work* 2:21–39 (1993) p. 22F.

91 Pamela H. Smith, Amy R. W. Meyers and Harold J. Cook (eds) *Ways of Making and Knowing: The Material Culture of Empirical Knowledge* (Ann Arbor: University of Michigan Press, 2014); Pamela H. Smith and Tonny Beentjes, ‘Nature and Art, Making and Knowing: Reconstructing Sixteenth-Century Life-Casing Techniques’ *Renaissance Quarterly* 63:1 (2010) 128–179 (p. 168 and p. 175).

92 Simon Werrett, ‘Preserving Nature: Domestic Thrift and Techniques of Conservation in Early Modern England’ *Notes and Records* 76 (2022) 317–329, p. 320. For more recent work in this area see Leonie Hannan, *A Culture of Curiosity: Science in the eighteenth-century home* (Manchester, Manchester University Press, 2023) and see also Simon Werrett, *Thrifty Science: Making the Most of Materials in the History of Experiment* (Chicago: University of Chicago Press, 2018).

93 Claire. L. Jones (ed.) *Rethinking modern prosthesis in Anglo-American commodity cultures, 1820–1939* (Manchester, Manchester University Press, 2017).

94 Steven Shapin, “The Invisible Technician,” *American Scientist* 77, no. 6 (1989): 554–563 (p. 562).

95 Susan. Leigh Star and Anselm Strauss, ‘Layers of Silence, Arenas of Voice: The Ecology of Visible and Invisible Work’ *Computer Supported Cooperative Work* 8: 9–30 (1999) p. 9. See also Shapin, Steven. “The Invisible Technician.” *American Scientis*t, 77: 6 (1989): 554–563.

96 Elaine Leong, ‘Collecting Knowledge for the Family: Recipes, Gender and Practical Knowledge in the Early Modern English Household’ *Centaurus* 55 (2013) 81–103. P. 95.

97 Coreen McGuire, ‘Who Invented the Possum? What Historians Can Learn from Disabled Innovation in Britain's Responaut Communities’ *Technology and Culture* 65:1 (2024): 89–116 (p. 109).

98 Hazel Morrison, ‘Constructing Patient Stories: “Dynamic” Case Notes and Clinical Encounters at Glasgow Gartnavel Mental Hospital, 1921–32’ *Medical History* 60:1 (2016) 67–86 (p. 85).

99 Kerry Davies, ‘Silent and Censured Travellers' Patients' Narratives and Patients' Voices: Perspectives on the History of Mental Illness since 1948’ *Social History of Medicine* 14:2 (2001) 267–292 (p. 272).

100 Sarah Holland, ‘Narrating and navigating patient experiences of farm work in English psychiatric institutions, 1845–1914’ in *Patient Voices in Britain, 1840–1948* edited by Anne Hanley and Jessica Meyer (Manchester: Manchester University Press, 2021).

101 Michael Worboys, ‘The non-patient's view’ in *Patient Voices in Britain, 1840–1948* edited by Anne Hanley and Jessica Meyer (Manchester: Manchester University Press, 2021) p. 36.

102 Michael Worboys, ‘The non-patient's view’ in *Patient Voices in Britain, 1840–1948* edited by Anne Hanley and Jessica Meyer (Manchester: Manchester University Press, 2021) p. 54.

103 Beth Linker and Emily K. Abel, ‘Integrating Disability, Transforming Disease History: Tuberculosis and Its Past’ in *Civil Disabilities: Citizenship, Membership, Belonging* edited by Nancy J. Hirschmann and Beth Linker (Philadelphia: University of Pennsylvania Press, 2015) 83–102 (p. 101).

104 William Viney, Felicity Callard, Angela Woods, ‘Critical medical humanities: embracing entanglement, taking risks’ *Medical Humanities* 41 (2015) 2–7 (p. 3).

105 Ludwik Fleck, *Genesis and Development of a Scientific Fact*. (Chicago: University of Chicago Press, 1979).

106 Gemma Almond-Brown, *Spectacles and the Victorians: Measuring, defining and shaping visual capacity* (Manchester, Manchester University Press, 2023) p. 88.

107 For example, see Charles Rosenberg. ‘Framing Disease: Illness, Society and History.’ In *Framing Disease: Studies in Cultural History*, edited by Charles E. Rosenberg and Janet Golden, xiii–xvi. New Brunswick, NJ: Rutgers University Press, 1992.; Geoffrey C Bowker and Susan Leigh Star, *Sorting Things Out: Classification and Its Consequences*. Cambridge, MA: MIT Press, 2000. Donald A Mackenzie, *Statistics in Britain 1865–1930: The Social Construction of Scientific Knowledge*. Edinburgh: Edinburgh University Press, 1981; Lorraine Daston, *Classical Probability in the Enlightenment*. Princeton: Princeton University Press, 1988; Ian Hacking*The Taming of Chance*. Cambridge: Cambridge University Press, 1990; Theodore M. Porter, *Trust in Numbers: The Pursuit of Objectivity in Science and Public Life*. Princeton: Princeton University Press, 1995; Theodore M. Porter, *Genetics in the Madhouse: The Unknown History of Human Heredity*. Princeton: Princeton University Press, 2018.

108 Coreen McGuire, *Measuring Difference, Numbering Normal: Setting the standards for disability in the interwar period* (Manchester, Manchester University Press, 2020).

109 Penny Richards and Susan Burch, ‘Documents, Ethics, and the Disability Historian’ in *The Oxford Handbook of Disability History* edited by Michael Remis, Catherine Kudlick and Kim E. Nielsen (Oxford, Oxford University Press, 2018) 161–174 (p. 162).

110 Elizabeth Newswald, ‘Things That Don't Talk Much and Things That Feel: Developing a Material Culture Methodology for “Black Box” Medical Devices’ *Nuncius* 35:3 (2020) 632–659 (p. 635).

111 Mara Mills, Jaipreet Virdi, and Sarah F. Rose, ‘Introduction’ to Special Issue on ‘Disability and the History of Science’ *Osiris* 39 forthcoming (2024).

112 Marion Schmidt, *Eradicating deafness? Genetics, Pathology, and Diversity in Twentieth-Century America* (Manchester, Manchester University Press, 2020).

113 Peter Dear, ‘What is History of Science the History of’ *Isis* 96:3 (2005) p. 406.

114 John Harley Warner, ‘The History of Science and the Sciences of Medicine’ *Osiris* 10 (1995) 164–193 (p. 165.).

115 ‘Prosthetic/artificial limbs used by Tracey Baynam’ 1967, Hanger Orthopaedic Group, Available on the Science Museum Website, https://collection.sciencemuseumgroup.org.uk/objects/co8594794/prosthetic-artificial-limbs-used-by-tracey-baynam-artificial-leg-prosthetic-leg.

116 Hilary Davidson, ‘Holding the Sole: Shoes, Emotions, and the Supernatural’ in Stephanie Downes, Sally Holloway and Sarah Randles, *Feeling Things: Objects and Emotions through History* (Oxford, Oxford University Press, 2018) 72–94 (p. 72).

117 Hilary Davidson, ‘Holding the Sole: Shoes, Emotions, and the Supernatural’ in Stephanie Downes, Sally Holloway and Sarah Randles, *Feeling Things: Objects and Emotions through History* (Oxford, Oxford University Press, 2018) 72–94; Susan M. Ward, ‘Does Anyone See Me? Playing Host to the Uninvited Guest of Post-Polio Syndrome’ *Qualitative Inquiry* 14:3 (2008) 360–383 (p. 376).

118 See R. P. Cheng MSW ‘Sociological Theories of Disability, Gender, and Sexuality: A Review of the Literature’, *Journal of Human Behavior in the Social Environment*, 19:1 (2009) 112–122 and R. Garland-Thomson, ‘Integrating Disability, Transforming Feminist Theory’ *NWSA Journa*l 14:3 (2002).

